# Application of next-generation sequencing in acute tonsillitis complicated with descending necrotizing mediastinitis: A case report

**DOI:** 10.1097/MD.0000000000038798

**Published:** 2024-07-05

**Authors:** Feng Zhao, Leqing Lin, Hui Wang, Lei Wang, Yuxuan Xu, Liang Guo

**Affiliations:** a Department of Intensive Care Unit, Affiliated Hospital of Hangzhou Normal University, Hangzhou, Zhejiang, China; b Emergency department, Litongde Hospital of Zhejiang Province, Hangzhou, Zhejiang, China.

**Keywords:** antibiotics, case report, descending necrotizing mediastinitis, next-generation sequencing

## Abstract

**Rationale:**

Descending necrotizing mediastinitis (DNM) is a rare but serious complication of oral and cervical infections that is associated with high mortality because diagnosis can be difficult or delayed. Early diagnosis and accurate identification of the causative pathogen can significantly reduce mortality, and are critical for the management of these patients.

**Patient concerns:**

A 56-year-old female was admitted with a sore throat and fever. The initial diagnosis was acute tonsillitis, but she was transferred to the intensive care unit after developing dyspnea.

**Diagnoses:**

Pleural effusion and mediastinal lesions were detected by computed tomography, and a diagnosis of DNM was confirmed by laboratory tests.

**Interventions:**

Initial treatment consisting of ceftriaxone and vancomycin with chest tube drainage were not effective. Thoracic surgery was performed to completely remove the “moss” tissue, blood clots, and pus. Next-generation sequencing was then performed, and the anti-infective treatment was changed to imipenem and linezolid based on these results.

**Outcomes:**

Eventually, the patient’s symptoms were controlled, all vital signs were stable, and she was successfully transferred out of the intensive care unit.

**Lessons:**

Next-generation sequencing is a rapid and accurate method for identification of pathogens that can provide a basis for early treatment of DNM, thereby improving patient prognosis and reducing mortality.

## 1. Introduction

Descending necrotizing mediastinitis (DNM) is a purulent mediastinal inflammation caused by a purulent infection in the oropharynx and deep neck due to entry of a pathogen through the cervical fascial space.^[1]^ It is a critical condition that progresses rapidly, is easily missed or misdiagnosed in its early stages, and has a mortality rate of 25% to 40%.^[2]^ These infections may originate in the odontogenic (36–47%), pharyngeal (33–45%), or cervical (15%) regions, or from other head and neck infections (5%).^[3]^ Estrera et al^[1]^ proposed diagnostic criteria for DNM in 1983, and these criteria have been used ever since. These criteria are: clinical manifestations of severe oropharyngeal infection; characteristic imaging findings; confirmation by intraoperative examination or autopsy; and clear source of oropharyngeal infection. The mortality rate is <20% when there is early and unequivocal diagnosis; early incision with adequate drainage and removal of necrotic tissue; targeted use of antibiotics; oral, maxillofacial, and thoracic surgery; and admission to an intensive care unit (ICU), infection department, or receipt of multidisciplinary comprehensive treatment.^[4]^

Here we report a case of acute tonsillitis with DNM in which the causative pathogens were detected by next-generation sequencing (NGS).

## 2. Case presentation

A 56-year-old female patient with an unremarkable medical history presented at our hospital with sore throat for 4 days and fever and chills for 1 day. One day before presentation, she went to a local clinic because of the gradual development of dysphagia. Her temperature at that time was 39.0 °C and she was given alozlocillin as anti-infective treatment, but reported no change in symptoms. She therefore went to our hospital’s infectious disease unit for treatment.

**Figure 1.**
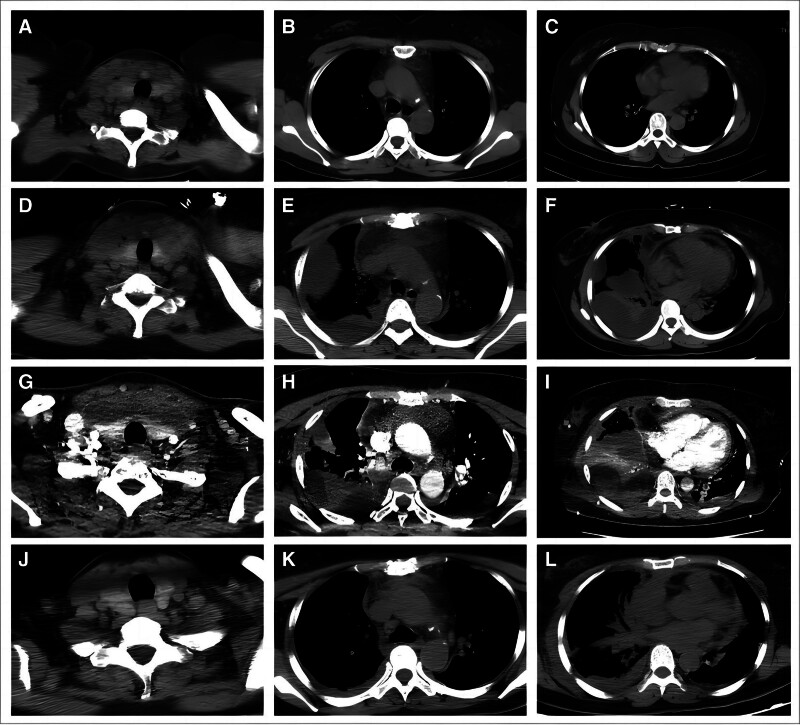
CT images of the patient on Day 0 (A–C), showing right cervical soft tissue swelling and punctate gas opacities, but no chest abnormalities; Day 3 (D–F), showing bilateral pleural effusion, right encapsulated effusion, pneumomediastinum, and pericardial effusion; Day 5 (G–I), showing bilateral pleural effusion, right encapsulated effusion, pneumomediastinum, and pericardial effusion; and Day 17 (J–L), showing decreased pleural effusion and mediastinal exudation, and absorption of pericardial effusion.

On admission, the physical examination results were tympanic temperature of 36.2 °C, respiratory rate of 18 bpm, radial artery blood pressure of 114/75 mm Hg, second-degree swelling in the right tonsil, purulent secretions, clear lung and breath sounds on auscultation, and no dry or wet rales. The laboratory results indicated elevated levels of white blood cells (WBCs,16.38 × 10^9^/L), neutrophils (91.4%), and C-reactive protein (CRP, 241.48 mg/L) (Table 1). A computed tomography (CT) scan of the chest showed swelling of the soft tissues of the right neck with punctate air accumulation, but no abnormalities in the chest cavity (Fig. 1A–C). At that time, the diagnosis was “acute purulent tonsillitis,” the patient received piperacillin/tazobactam as empirical treatment for infection, and a blood sample was taken for culture.

**Table 1:** Vital signs and laboratory results from Day 0 (initial presentation) to Day 17.

Index	Day 0	Day 3	Day 5	Day 17
Tympanic temperature, °C	36.2	38.6	38.4	36.4
Oxygen saturation, %	99	93	95	100
Respiratory rate, bpm	18	40	26	18
Blood pressure, mm Hg	114/75	149/96	139/68	118/68
WBCs, ×10^9^/L	**16.38**	**13.21**	**13.41**	6.04
Neutrophils, %	**91.4%**	**86.5%**	**82.8%**	73.4%
CRP, mg/mL	**241.48**	**230.27**	**176.75**	**22.02**
PCT, ng/mL	NA	**1.40**	**0.75**	<0.10
IL-6, pg/mL	NA	**92.80**	**162.40**	**15.99**

A bold value indicates that it is out of the normal range.

Abbreviations and reference ranges: CRP = C-reactive protein, 0 to 10 mg/L, IL-6 = interleukin 6, <7.0 pg/mL, neutrophils = 40% to 75%, PCT = procalcitonin, 0 to 0.5 ng/mL, WBCs = white blood cells, (3.5–9.5) × 10^9^/L.

On the third day after admission, she developed chest tightness and shortness of breath. After administration of oxygen *via* nasal catheters (flow rate: 3 L/min), the blood oxygen saturation was 93%. Pulmonary auscultation demonstrated that breathing through the right lung was poor and had a moderate wet rattle, and the left lung had mild dry rhonchi. A second thoracic CT showed bilateral pleural effusion, right encapsulated effusion, gaseous exudation in the upper mediastinal fat space, and pericardial effusion (Fig. 1D–F). Laboratory results at that time indicated elevated levels of WBCs (13.21 × 10^9^/L), neutrophils (86.5%), CRP (230.27 mg/L), and procalcitonin (PCT, 1.40 ng/mL) (Table 1). The blood culture from 3 days earlier was negative. Considering that she had a severe lung and mediastinal infection, she was transferred to the ICU for further treatment.

The ICU immediately adopted an oxygen therapy strategy of alternating high-flow oxygen inhalation and noninvasive ventilation. They also performed a thoracentesis using an indwelling chest tube and collected pleural fluid for bacterial culture. Because there was no clear evidence of the etiology, ceftriaxone and vancomycin were administered as an empirical treatment. On the third day in the ICU and receipt of oxygen therapy, her blood oxygen saturation was basically in the normal range, but her tympanic temperature remained high. The laboratory test results at that time indicated elevated levels of WBCs (13.41 × 10^9^/L), neutrophils (82.8%), CRP (176.75 mg/L), and PCT (0.75 ng/mL) (Table 1). An enhanced CT at that time showed bilateral pleural effusion, right-sided encapsulated effusion, exudative pneumatisation of the upper mediastinal fat space, and pericardial effusion, similar to the previous results (Fig. 1G–I). The cultures of sputum, blood, and pleural fluid remained negative. The otolaryngologist performed a laryngoscopy, and found right peri-tonsillitis secondary to parapharyngeal, retropharyngeal, and mediastinal infection and abscess. The thoracic surgeon therefore performed closed thoracic drainage, and the anti-infective treatment was adjusted to ceftriaxone and linezolid.

On the fourth day in the ICU, a multidisciplinary team changed the anti-infective treatment to imipenem and linezolid, and decided to proceed with surgical treatment. On the fifth day in the ICU, thoracic surgeons performed a thoracotomic examination with a mediastinal incision and neck drainage and incision. During the operation, they saw adhesions in the right chest cavity, coverage of the lung surface with purulent “moss,” a “fiber-board” formation, local purulent secretion, and blood clots, with clear evidence of anterior mediastinal edema and formation of a purulent cavity. One portion of the surgically removed tissue was sent to the laboratory for NGS, and another portion was sent for bacterial culture. The NGS results indicated *Prevotella, Streptococcus, Pyramidobacter*, and other pathogens (Table 2). Following this result, the anti-infective treatment (imipenem and linezolid) was maintained and postoperative drainage, maintenance of circulatory stability, intensification of nutritional support, and other treatments were continued.

**Table 2:** Results of next-generation sequencing of drainage fluid collected on Day 5 in the ICU.

Bacteria				
Type^a^	Genus	Sequences (n)	Species	Sequences (n)
G^−^	*Prevotella*	1635	*P. oris*	584
			*P. buccae*	399
G^+^	*Streptococcus*	216	*S. constellatus*	73
			*S. anginosus*	3
G^−^	*Pyramidobacter*	143	*P. piscolens*	143
G^−^	*Porphyromona*	68	*P. endodontalis*	63
			*P. gingivalis*	4
G^−^	*Treponema*	36	*T. medium*	14
			*T. denticola*	10
Fungi, viruses, parasites: no detected sequences				
*Mycobacterium tuberculosis, Mycoplasma, Chlamydia, Rickettsia*: no detected sequences				

a G^+^: Gram-positive bacteria, G^−^: Gram-negative bacteria.

On the eighth day in the ICU, *Staphylococcus epidermidis* was identified in bacterial culture of the blood. Drug susceptibility testing indicated that the current treatment was suitable, so this treatment was maintained. On the tenth day in the ICU, the patient reported no chest tightness or shortness of breath, and only a small amount of yellow fluid was drained from the chest and mediastinal drainage tubes. A bedside ultrasound at that time showed pleural effusion of only 1 cm, so the chest and mediastinal drainage tubes were removed.

**Figure 2.**
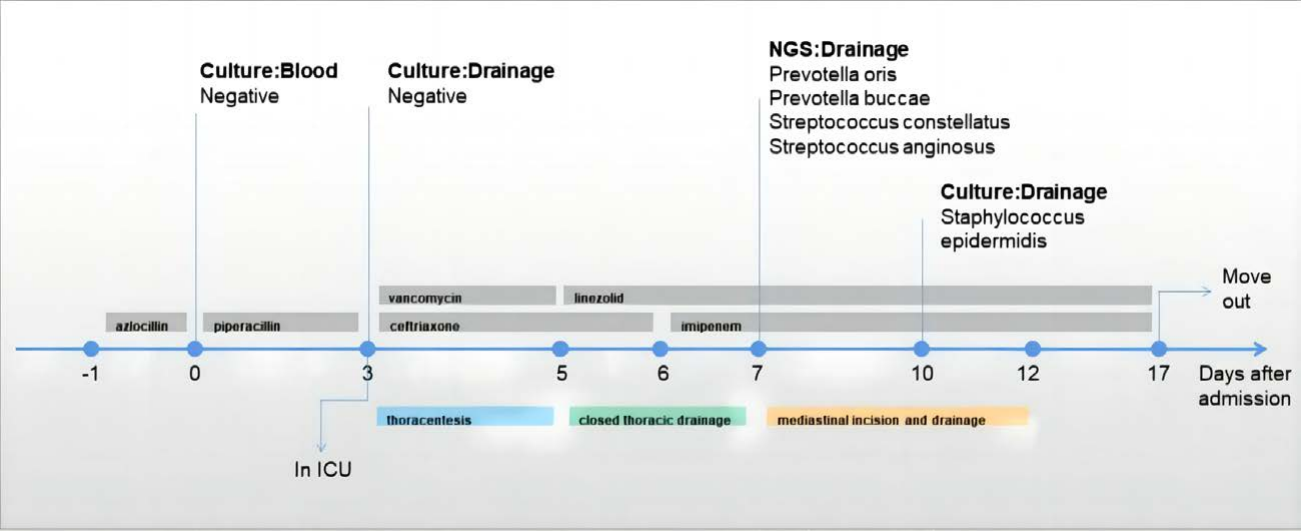
Timing of different treatments and collection of samples for culture and NGS. NGS = next-generation sequencing.

On the 15th day in the ICU, the patient had stable oxygenation with the nasal catheters in place, no shortness of breath, and a normal tympanic temperature. At that time, the laboratory tests showed substantial normalization, with a WBC count of 6.04 × 10^9^/L, 73.4% neutrophils, a CRP of 22.02 mg/L, and a PCT below 0.10 ng/mL (Table 1). A third thoracic CT showed diminished pleural effusion, and absorption of the mediastinal exudation and pericardial effusion (Fig. 1J–L). Therefore, we transferred the patient from the ICU to the general ward of the thoracic surgery department and continued treatment, with the details described in Figure 2. During her hospitalization in the thoracic surgery department, the anti-infective treatment regimen of imipenem plus linezolid was continued, and her condition improved further. Her body temperature remained in the normal range, the inflammatory markers gradually decreased to the normal range, and she reported no symptoms, such as sore throat, tightness in the chest, shortness of breath, or fatigue. On 29 days after the initial presentation at our hospital’s infectious disease unit, she was discharged with a smile on her face.

At 1, 2, and 5 months after discharge, she returned to the hospital for follow-up examinations with high-resolution CT of the chest. The results showed no oozing or pneumatosis in the mediastinum and no enlarged lymph nodes. The patient reported that she was slowly returning to her usual rhythm of life.

## 3. Discussion

DNM is a serious complication of oral, pharyngeal, and cervical infections that can rapidly progress to sepsis and death. Several anatomical and physiological factors facilitate the spread of oral and pharyngeal infections and other infections deep into the neck and the mediastinum,^[5]^ including the presence of a large amount of loose connective tissue in the face and neck, the spontaneous inspiratory movement of the patient, and gravitational force and the negative pressure environment of the chest cavity. The mediastinum mainly consists of loose connective tissues that are rich in fat and lymphatic tissue. Once infected, the pathogen and associated inflammation can spread to all the mediastinal organs, resulting in mediastinal effusion, production of pus, and empyema, all of which can lead to the rapid onset of sepsis and septic shock, and ultimately to multiple organ failure and death.^[3]^

DNM can occur between 12 hours and 14 days after a neck infection, most typically within 48 hours of an infection. The early clinical manifestations are insidious, but may these occur after the infection has spread.^[6]^ These manifestations include swelling and pain in the neck and anterior chest, dysphagia due to abscess compression of the esophagus, dyspnea due to mediastinal and thoracic pus accumulation, circulatory disturbances due to abscess compression of the large blood vessels in the thoracic cavity, and systemic symptoms such as fever and sepsis. According to Endo et al^[7]^ classified DNM as type I (infection confined to the anterior mediastinum in the plane of the tracheal ramus), type Iia (spread of infection to the anterior inferior mediastinal space), and type Iib (spread of infection to the anterior and posterior mediastinum). More recently, Sugio et al^[8]^ proposed classification of an infection confined to the posterior mediastinum as type Iic, in which a more extensive infection was associated with a higher mortality rate.^[9]^

The rarity and severity of DNM, and the tendencies for delays in diagnosis and treatment are the major reasons for the high mortality rate of these patients.^[10]^ Due to the high mortality rate and rapid progression of DNM, early and accurate diagnosis, timely and adequate surgical drainage, targeted antibiotic therapy, and treatment in an ICU are critical for achieving successful treatment and rehabilitation.^[4]^ In recent years, minimally invasive approaches were recognized as the first choice for draining the pleural cavity and mediastinum.^[11]^ Additional evidence suggested that the application of vacuum sealing drainage (VSD) in cervical incision did not improve prognosis, but may shorten the length of ICU and hospital stays.^[12]^ CT is the best method for the early diagnosis of DNM. The typical findings include mediastinal widening or increased mediastinal fat density (100%), focal or diffuse gas in the mediastinum (19–54%), gas–liquid level (30–55%), and pleural effusion (67–85%). The other manifestations include pericardial thickening, effusion, mediastinal lymphadenopathy, and pulmonary infiltration.^[3,9]^ CT also plays an important role in monitoring the drainage process, guiding treatments, and evaluating therapeutic effects.^[13]^

The identity of the causative pathogen is another major factor affecting the clinical prognosis of patients with DNM. These pathogens are mostly a mixture of bacteria from the oral cavity, pharynx, and neck.^[6]^ The most common microorganisms include *Prevotella, Clostridium, Veronococcus*, and *Streptococcus oralis*,^[5]^ and less common microorganisms are *Pseudomonas aeruginosa, Enterobacter, Mycobacterium*, and *Klebsiella pneumoniae*. However, identification of the causative pathogen by in vitro culture is often time-consuming and cumbersome, and most pathogens cannot be successfully cultured.^[14,15]^ A previous study estimated that the causative pathogen was not isolated by culture in approximately 40% of DNM cases.^[16]^ Therefore, a rapid, specific, and high-throughput detection method is essential for the diagnosis and timely control of the pathogens responsible for DNM and other infectious diseases.

NGS is a powerful tool for detection of pathogens, and can identify many thousands of pathogens in approximately 16 to 24 hours. In addition to identification, NGS also provides information on the distribution and proportion of different pathogens in a clinical sample. The basic principles of NGS are extraction and fragmentation of nucleic acids from a sample, preparation of a library of nucleic acid sequences, parallel sequencing of the reads, and identification of species in the metagenome by analysis with a database. Thus, the NGS process consists of wet-lab procedures (sample pretreatment, nucleic acid extraction, building a library) and dry lab procedures (data quality control, human sequence removal, alignment of microbial sequences, and analysis of genes related to drug resistance or virulence).^[17,18]^ Chen et al^[19]^ compared NGS with traditional bacterial culture techniques for detecting pathogenic bacteria in the maxillofacial space. NGS had an average detection time of 18.81 ± 3.73 hours and a positive detection rate of nearly 100%; conventional bacterial culture had an average detection time of 83.25 ± 11.64 hours and a positive detection rate of only 31.25%.

Our patient had DNM due to acute tonsillitis (a condition only rarely associated with DNM) and we identified *Staphylococcus epidermidis* by blood culture 8 days after ICU admission; however, we could not exclude the possibility that this species was the result of a nosocomial infection and not the actual cause of DNM. NGS can rapidly detect all pathogens in a sample of infected tissue, and we detected 2098 sequences from the patients’ drainage fluid, mostly from *Prevotella oris* (n = 584, 27.84%), *Prevotella buccae* (n = 399, 19.02%), *Pyramidobacter piscolens* (n = 143, 6.82%), and *Streptococcus constellatus* (n = 73, 3.48%). This rapid detection facilitated the timely adjustment of anti-infective treatment, early surgical drainage, and multidisciplinary care in the ICU, and led to a favorable outcome.

## 4. Conclusion

In daily practice, DNM should be suspected in a patient who presents with oral and deep throat infections with fever, chest pain, dysphagia, and dyspnea. CT scans should be performed as soon as possible to clarify the diagnosis and ensure timely treatment. At the same time, given the limitations of traditional culture methods, NGS should be considered for routine testing because it is inexpensive and provides rapid and comprehensive results, thereby allowing the timely treatment of DNM patients and improving clinical prognosis.

## Acknowledgments

We thank Medjaden Inc. for scientific editing and proofreading of this manuscript.

## Author contributions

**Writing – original draft:** Feng Zhao.

**Writing – review & editing:** Leqing Lin, Liang Guo.

**Formal analysis:** Hui Wang, Lei Wang, Yuxuan Xu.

**Conceptualization:** Liang Guo.
